# Intersectionality Research for Transgender Health Justice: A Theory-Driven Conceptual Framework for Structural Analysis of Transgender Health Inequities

**DOI:** 10.1089/trgh.2019.0039

**Published:** 2019-10-29

**Authors:** Linda M. Wesp, Lorraine Halinka Malcoe, Ayana Elliott, Tonia Poteat

**Affiliations:** ^1^ University of Wisconsin-Milwaukee College of Nursing, Milwaukee, Wisconsin.; ^2^ University of Wisconsin-Milwaukee Joseph J. Zilber School of Public Health, Milwaukee, Wisconsin.; ^3^ The Elliott Group LLC, Los Angeles, California.; ^4^ Center for Health Equity Research, UNC School of Medicine, University of North Carolina at Chapel Hill, Chapel Hill, North Carolina.

**Keywords:** health inequity, health justice, intersectionality, structural determinants of health, structural injustice, transgender

## Abstract

Transgender people experience intersecting forms of social marginalization and are disproportionately affected by health inequities. We elucidate a novel conceptual framework for transgender health research that theorizes the constructs and pathways through which social inequities produce health inequities for transgender populations. Drawing on theories of intersectionality and structural injustice, Intersectionality Research for Transgender Health Justice (IRTHJ) posits that social and health inequities affecting transgender populations are the result of status quo power relations produced within and between oppressive structures, institutional systems, and socio-structural processes. The IRTHJ framework delineates three main actions for improving transgender health research: (i) name intersecting power relations, (ii) disrupt the status quo, and (iii) center embodied knowledge. The authors show how IRTHJ provides tools for researchers to transform the design, implementation, and interpretation of transgender health research, and they discuss implications for programs, policy, and action for transgender health justice.

## Background

Transgender^‡^ people globally experience intersecting forms of social marginalization and are disproportionately affected by health inequities.^1^ Despite greater ill health, transgender people face significant barriers in accessing health care due to discrimination, harassment, and refusal of care from health care providers.^2^ Among the 27,715 respondents to the 2015 United States Transgender Survey, 24% reported past-year transgender-related housing discrimination and 30% of employed respondents reported job-related discrimination, with transgender people of color reporting even higher rates.^2^ Sex work is often a means of survival for transgender people, and transgender women sex workers are vulnerable to HIV because of a complex interaction of systemic, interpersonal, and individual discrimination and violence.^3^ Worldwide, HIV is higher among transgender women when compared with all adults of reproductive age,^4^ and in the United States, African American transgender women experience a disproportionately higher burden of HIV.^5^ The United States National Institutes of Health has identified transgender people as a health disparity population, due to their disproportionate burden of mental and physical ill health,^6^ and the World Health Organization has called for a global health agenda inclusive of transgender people.^7^

Transgender populations face significant inequities in violence and mental health concerns. In the United States, compared with non-transgender people, transgender people had disproportionately higher prevalence of suicide attempts^8^ and substance use,^9^ with even higher rates among transgender people of color and transgender people with disabilities.^2^ Estimates suggest that United States Black and Latina transgender women account for nearly 93% of all transgender homicide victims, and that these women are murdered at higher rates than Black and Latina cisgender women.^10^

Transgender health research requires a theoretical foundation that can inform inquiry into relationships among these layers of marginalization, discrimination, and health inequities. The Syndemic theory has been utilized in the transgender health literature to analyze social factors that underlie co-occurring health outcomes.^11–13^ However, a recent review of HIV and related syndemics among transgender populations, which found that multiple co-occurring psychosocial conditions were associated with HIV risk, called for future research to employ a more complex analysis of structural-level factors that create and sustain these conditions.^14^

## Purpose

This article elucidates a new theory-driven conceptual framework for research on transgender health inequities. Drawing on intersectionality^15^ and structural injustice,^16^ we theorize the constructs and pathways through which social inequities produce health inequities for transgender populations. We outline key theoretical tenets and provide actions and tools for researchers to elucidate the structural production of transgender health inequities, thereby producing intentional and actionable research that can transform inequities into transgender health justice. We are clinicians, researchers, and public health scholars of transgender and cisgender experience who, throughout this article, aim at demonstrating that remaining aware of all aspects of our social positions within these systems and structures is a crucial component of this work.

## Theoretical Foundation

### Intersectionality

Intersectionality theory asserts that various oppressions create and mutually constitute one another to sustain a complex matrix of power that is rooted in, and actively maintained by, social structures and institutional systems.^15,17^ Intersectional thinking originated within various women of color resistance movements from the 19th and 20th centuries, including abolitionists Maria Stewart and Sojourner Truth, and the Black feminist lesbian organization Combahee River Collective.^18^ As well, early Indigenous feminists, such as Zitkala-Sa in the late 1800s, fought against violence that “has always been gendered, aged, and linked to access to land.”^19^ Crenshaw^20,21^ first coined the term “intersectionality” in her early scholarship on the complexity of oppression, which emphasized a shift in analytical thinking about relationships between and within the social categories of race and gender. Likewise, Collins described a “matrix of domination,” arguing that power cannot be reduced to only one oppressive structure or one dominant group, but instead functions as an “intangible entity that circulates within a particular matrix” of intersecting oppressions.^22^

Queer and transgender activists have for some time emphasized the importance of seeking justice through intersectional resistance that prioritizes multi-axis analysis, such as disrupting institutional systems that perpetuate racialized-gendered violence.^23^ Yet, although intersectionality has been applied to health research,^17,24–27^ seldom has it informed research with transgender populations.^28^ Next, we outline four tenets of intersectionality that are most applicable to understanding transgender health inequities.

First, an intersectional analysis attends to multiple intersecting structures of domination, such as cisgenderism, heteropatriarchy, white supremacy, and colonialism.^17^ Cisgenderism is the “cultural and systemic ideology that denies, denigrates, or pathologizes self-identified gender identities that do not align with assigned gender at birth as well as resulting behavior, expression, and community.”^29^ Pertinent to cisgenderism is Connell's argument that gender structures are macro-level patterns found across institutions worldwide that are socially embodied, dynamic, and highly influenced by colonial processes over the past 500 years.^30^ Gender hierarchies, in concert with other structures of power that interlock and sustain social inequities, shape intersecting social identities and experiences at the micro level.^25^ For example, intersectionality research on high rates of sex work, HIV, and incarceration among Black transgender women might consider how systemic anti-Black racism, heteropatriarchy, and transphobia operate within a capitalist economy to produce housing segregation, mass incarceration, precarious employment, and extreme vulnerability to violence for this population, as well as how these structures perpetuate the objectification and criminalization of Black femininity.^31^

Second, intersectionality elucidates how structures of domination manifest through “processes of differentiation,” which create categories of both difference and identities marked as different by the dominant group.^17^ Intersectionality-informed health inequities research is explicit in defining which of these concepts (i.e., processes, categories, identities) are being studied and why, as well as how they are rooted in structures of domination.^25,32^ Examining processes of differentiation (e.g., gendering, racialization, pathologizing) draws attention to the ideologies embedded in structures of domination that inform societal norms and standards.^17^ Categories of difference (e.g., gender, race, social class, ability) are the result of these everyday social processes that demarcate groups as “other” and create and sustain power differentials between them.^17,21^ Jones argues that a category of difference, such as race, is a proxy for racism (a structure of domination), not a biological marker of innate differences.^33^ Roberts further explains race as produced by intersections of racism and capitalism by emphasizing how scientific, political, and capitalist endeavors work together to perpetuate ideologies and market goods based on the false idea that race is based in genetics.^34^ Thus, constructs such as gender or race are not considered unidimensional or naturally occurring properties of individuals, but instead a result of the active production of socially marked and intersecting categories of difference.^32,35^

A pertinent example of this second tenet concerns racialized gender norms. As Spade and Willse explain, racialized gender norms “govern sexual behavior, speaking styles, diet, emotional range, punctuality, manners, dress, and much more. Discourses in the social and medical sciences, popular media, criminal and immigration systems, education, and social services industries produce and uphold these norms.”^36^ Applying intersectionality, transgender health researchers might examine discourses and other practices that (re)produce these norms, along with how they are enforced and internalized.

Third, intersectionality posits that social inequities are reflections of power differentials that negatively impact people with marginalized identities while privileging dominant groups.^32^ Intersectional health scholars, thus, emphasize the need to understand social categories as relational and indicative of intersecting power relations.^17,37,38^ For example, health disparities researchers might compare transgender women of color to white transgender women by conceptualizing and interpreting identities derived from the social categories of race and gender as effects of structurally produced power relations tied to racism and cisgenderism.^17^ Likewise, intersectionality informed research clarifies that categories considered normative (e.g., “white” or “cisgender”) are also those with more social power.^32,36^

Fourth, intersectionality centers the embodied knowledge of people who experience and resist multiple intersecting oppressions.^39^ Within intersectional scholarship, embodied knowledge “emphasize[s] and celebrate[s] the voices, experiences, situated knowledge, and perspectives of those traditionally marginalized and erased.”^17^ An intersectionality informed approach values embodied knowledge, because it is essential for meeting social justice aims, including understanding how processes of oppression work and how to resist them, and requiring researchers to be accountable to people who live in the margins of society.^39^ For example, scholars interested in researching transgender health inequities related to incarceration could begin by learning about specific experiences of transgender prisoners, as depicted in anthologies such as *Captive Genders: Trans Embodiment and the Prison Industrial Complex*,^40^ written by current and former transgender prisoners, activists, and academics. These writings are a collection of experiences and plans for action, offering an analysis of the ways that “regimes of normative sexuality and gender are organizing structures of the prison industrial complex.”^40^ However, this is only a start; fully centering embodied knowledge requires participatory research approaches where people situated at the intersection of multiple oppressed identities are included as decision makers in research and knowledge production.^39,41^

### Theory of structural injustice

Green et al. state that “intersectionality will be most effective where combined with social theory on the production of health inequalities.”^42^ Young's theory of structural injustice provides a framework for understanding how unjust social processes have become the status quo and create and maintain social inequities via institutional systems.^16^ The theory of structural injustice posits that individuals act according to societal laws, rules, and other accepted practices that operate together as the “norm” to directly benefit some, while indirectly or directly harming others.^16^ As Young states, the “structures are produced and reproduced by large numbers of people acting according to normally accepted rules and practices, and it is in the nature of such structural processes that their potentially harmful effects cannot be traced directly to any particular contributors.”^16^ The theory of structural injustice identifies the status quo itself as an oppressive vehicle of power. Institutions are held in place by laws, implicit social rules, perceived advantages, or simply by nature of habit, and institutional systems appear objective and permanent, in part because the individuals within them follow standard operating procedures.^16^ Applying Young's^16^ theory to social inequities in health, researchers are directed to consider how the status quo functions and ways that everyday practices of multiple institutional systems (re)produce inequities.

Young^16^ further asserts that when we understand injustice as structural, that is, the result of various institutional systems and socio-structural processes in which we participate, it leads us, morally, to address our collective responsibility for justice. Young argues that the responsibility for justice lies with each person in society, even though each individual person may not be at fault. Responsibility is essentially shared and “can be discharged only through collective action.”^16^ Collective action, according to Young, is a forward-looking responsibility that manifests through actions that are intent on changing interrelated institutions and processes to make them more just. No single person can change the structures, systems, or processes on their own. Thus, research informed by the theory of structural injustice advances an ethics that encourages many people at a wide variety of positions within institutions and society to take collective responsibility for problematizing, analyzing, and disrupting the structures of injustice.

## Intersectionality Research for Trans Health Justice

Drawing on the theories of intersectionality and structural injustice, Intersectionality Research for Transgender Health Justice (IRTHJ) provides tools for researchers to transform the design, implementation, and interpretation of transgender health research, as well as to address implications for programs, policy, and action. IRTHJ assumes that social inequities and the distribution of societal determinants of health are not random or accidental, but rather are structural injustices that have been systematically produced over time through actively maintained structures of power. The main tenet of IRTHJ is that social and health inequities affecting transgender populations are the result of status quo power relations produced within and between oppressive structures, institutional systems, and socio-structural processes.

### IRTHJ conceptual framework

**FIG. 1.**
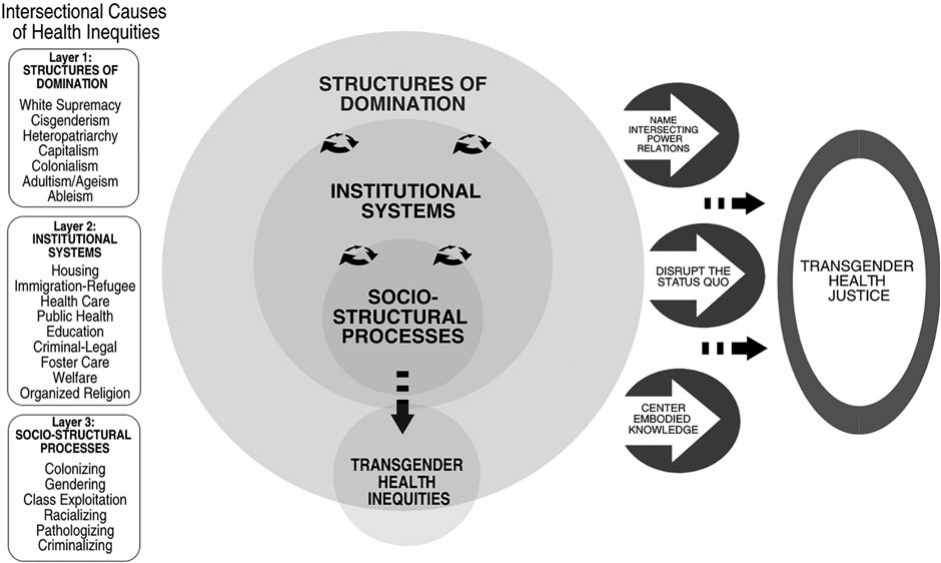
Intersectionality Research for Transgender Health Justice (IRTHJ) Framework.

The IRTHJ framework is illustrated in Figure 1. Three main layers, depicted as concentric circles, demonstrate how relations of power (structures of domination, institutional systems, and socio-structural processes) work together in a continuous interlocking manner, to produce transgender health inequities.

Structures of domination (Fig. 1, Layer 1) include the various forms of structural oppression and underlying ideologies that, we argue, ultimately cause social health inequities for transgender populations. Social structures involve “a way of looking at the whole society, one that sees patterns in relations among people and the positions they occupy relative to one another.”^16^ When considered through an intersectionality lens, transgender populations are seen to experience heightened vulnerabilities because of their structurally produced marginalized social positions at the intersections of race, ethnicity, citizenship, gender, sexuality, age, disability, and class.

Structures of domination are enacted through, and reinforced by, institutional systems (Fig. 1, Layer 2), which converge to create structural injustice for transgender populations. For example, cisgenderism permeates every institution in the United States, conferring privileges to those classified as cisgender while penalizing, pathologizing, and erasing transgender populations.^43^ These systems intersect in the policies, regulations, and laws that perform state surveillance and regulation of gender identification.^44^ A transgender person often must navigate both the health care and legal systems to change gender markers on their birth certificate, driver's license, and passport, because some states or jurisdictions require medically certified documentation confirming that an individual has undergone or is undergoing gender transition through cross-gender hormones and/or surgery.^44^ In addition to being onerous and dehumanizing, such policies restrict options for those who do not desire or cannot access hormones or surgery, and for people whose bodies are disproportionately criminalized because of interlocking oppressive structures such as racism and ableism.^44^

Socio-structural processes (Fig. 1, Layer 3) are everyday social practices that (re)produce inequality in service of the status quo by, for example, enforcing norms within and across institutional systems. Sevelius' study of self-identified transgender women of color found that they faced societal norms and expectations about femininity shaped by racism and sexism, which led to experiences of objectification, victimization, and identity threat.^31^ Transgender identities are pathologized through use of a medical diagnosis (gender dysphoria) that is still widely categorized as a mental health condition, yet is required for accessing various health care services.^45^ In addition, interventions such as hormones and surgery are often only obtainable when someone has the economic means to access a lengthy diagnostic process.^45^

### IRTHJ actions

**FIG. 2.**
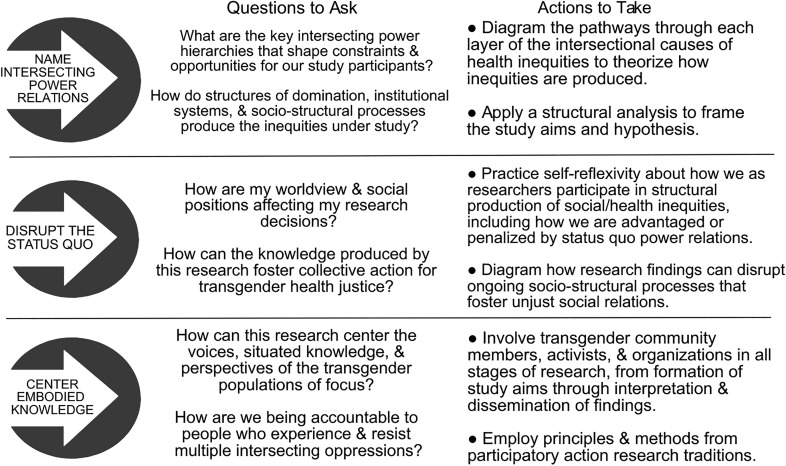
IRTHJ actions.

IRTHJ identifies three actions to advance transgender health justice. Figure 2 translates these actions into specific questions and steps for implementation.

First, naming intersecting power relations requires that researchers consider the structural production of transgender health inequities within and across each layer of the IRTHJ framework. At all stages of research—from initial study framing and development of research questions, to methods and analysis, and through interpretation and dissemination of findings—researchers can ask how the interlocking structures of domination, institutional systems, and socio-structural processes produce the transgender health inequities under study. IRTHJ requires researchers to theorize intersecting relations of power at multiple layers of analysis. An excellent example of this action in practice is Sevelius' Gender Affirmation Framework,^31^ wherein diagrams show how the intersecting experiences of racism, transphobia, and sexism create internalized stigma leading to an increased need for gender affirmation; at the same time, these structures of domination influence institutional systems that restrict access to the resources needed for gender affirmation.

Second, researchers seeking health justice disrupt the status quo by self-reflexivity and action toward building health equity. Power relations can unintentionally influence our language choices, study measures, analysis variables, and suggestions for policy interventions.^46^ In concert with Young's^16^ theory of structural injustice, researchers have a responsibility to disrupt hierarchies of power. Reflexivity requires researchers to analyze our everyday participation in the structural production of social and health inequities, including how we are advantaged or penalized within various institutions by status quo power relations. Reflexivity also calls on researchers to take account of the epistemological and ontological underpinnings that inform our worldviews, as well as how our intersecting social positions may impact our decisions throughout the research process.^47,48^ Disrupting the status quo is especially important when conducting community engaged research, where hierarchies and marginalization are often reproduced in the process of engaging community members as peer researchers and may cause more direct harms if not addressed.^49^

Here we share with readers a bit about our experiences and positionalities that have influenced our thinking and approach to generating this framework. L.M.W. identifies as a white cisgender woman from a working-class background, a nurse researcher, and a nurse practitioner with more than 15 years of experience working with transgender youth and adults in urban U. S. settings. L.H.M. identifies as a cisgender woman who is racialized as white and was reared in a U. S. working-class, biracial/biethnic (Ashkenazi Jewish and Black) family; she is a social epidemiologist with more than 20 years of experience in conducting health inequities research that draws on community knowledge as well as critical race, intersectional, and decolonial scholarship. A.E. identifies as a Black woman with some lived transsexual experience and from a working middle-class background, a doctorally prepared nurse practitioner, advocate, policy developer, and researcher with expertise in comprehensive care for transgender adults for 12 years. T.P. identifies as a Black queer woman from a working-class background; she has provided medical care for transgender adults for more than 20 years and has conducted research with transgender communities for 8 years. Collectively, we seek to employ IRTHJ in our work and carefully consider how our research activities will disrupt status quo power relations to advance health justice for transgender populations.

Third, centering embodied knowledge calls on researchers to value and be accountable to—in all research activities—the voices, situated knowledge, and perspectives of transgender populations, especially those who experience and resist multiple intersecting oppressions. Centering embodied knowledge is an acknowledgement that the margins of society “have been both a site of repression and a site of resistance.”^50^ This approach argues that confronting the intersecting structural and institutional harms experienced by multiply marginalized groups has the greatest potential for eliminating power hierarchies, thereby producing social and health equity for all.^51^ Participatory research approaches that are grounded in collaborative relationships with transgender communities are most effective for centering embodied knowledge. These approaches emphasize trust-building as a continuum, while acknowledging historically unjust relationships between marginalized communities and researchers.^52^ To center embodied knowledge, researchers can draw from core principles of community-based participatory research and participatory action research, such as building meaningful relationships, acknowledging and sharing power, encouraging participation, privileging the community's knowledge, and making equity-oriented change.^53^

## Discussion

IRTHJ is designed to be applied broadly across an array of transgender populations. In this section, we apply IRTHJ to three previously published studies researching HIV among transgender women, all of whom represent communities situated at the intersection of multiple oppressed identities. We illustrate how the IRTHJ framework can help illuminate the ways these studies have already uncovered some of the structural causes of transgender health inequities, and we discuss how use of IRTHJ can strengthen future research and interventions.

We begin by briefly describing the three studies. Brennan et al. were the first to apply the syndemic theory to investigate HIV risk among young transgender women.^12^ The authors conducted a secondary data analysis of a cross-sectional study of transgender women (15–24 years) in the Chicago and Los Angeles metro areas. They explored whether there was an “additive” or dose–response relationship between four syndemic factors and two study outcomes: condomless anal intercourse and self-reported HIV serostatus. They also examined associations between their syndemic index and three indicators of social marginalization. Participants (*n*=151) reported their race/ethnicity as Black/African American (39%) or Latina (38%), and 34% were employed. The findings showed positive associations between two syndemic factors (intimate partner violence and polysubstance use) and the study outcomes, and an association between two indicators of social marginalization (sex work and incarceration) and the syndemic index. The authors concluded with a call for a “multisystems approach” to prevention that targeted “both social and health-related factors.”^12^

Wilson et al. also applied the syndemic theory to a cross-sectional survey of young (ages 16–24) transgender women (*n*=282) in the San Francisco area; they examined racial/ethnic differences in sociodemographic factors, including racial discrimination, HIV-related risk behaviors, and syndemic factors.^13^ Among the participants, 36.8% were white, 21.9% Latina, 15.2% mixed race, 13% African American, 5.9% Asian, and 7.1% “other.” The investigators collapsed the sample into two groups, “white” and “racial/ethnic minority.” They found significantly lower education and significantly greater levels of racial discrimination (but nearly identical levels—80%—of trans-related discrimination), greater childhood housing instability, and greater engagement in condomless receptive anal intercourse (CRAI) among racial/ethnic minorities compared with whites. The article concluded with a call for prevention efforts that address “racial inequalities to reduce stressors” and “macrolevel disparities” particularly impacting young transgender women from racial/ethnic minority groups.

Utilizing a gender minority stress framework, Arayasirikul et al. examined transphobic discrimination and “race” in relation to two HIV risk factors (binge drinking and CRAI) among HIV-negative trans women (*n*=149) in San Francisco.^54^ In bivariate analyses, the authors compared white trans women with “transwomen of color”—the latter included Black (22.8% of sample), Latina (33.6%), and “other” (18.1%) trans women. Results showed that white trans women were disproportionately older, college educated, U. S. born, insured, and reported more transphobic discrimination measures (e.g., ever been denied housing or evicted because of gender identity or presentation). In multivariate analyses, higher levels of transphobic discrimination were associated with recent binge drinking but not with CRAI; trans women of color status (used synonymously with “race”) was associated with CRAI but not with binge drinking. The authors concluded that “interventions informed by racial equity and social justice to specifically target trans women of color may be effective in preventing [C]RAI.”

### Name intersecting power relations

The three papers do important work to examine how social factors (e.g., homelessness, discrimination, sex work) and social stressors (e.g., victimization, parental rejection) are associated with health outcomes, providing a dual focus on psychosocial and social determinants of transgender health inequities. Using the IRTHJ framework, we go further to ask why transgender populations have a worse distribution of these social determinants. IRTHJ calls for theorizing, through each IRTHJ layer, the intersecting structural causes of the social patterning of study measures. For example, looking to layer 3 of Figure 1, research that names social-structural processes (such as class exploitation, racialization, and criminalization) leads to deeper understanding about why transgender women of color have an increased vulnerability to homelessness, incarceration, and sex work, as Sevelius highlighted.^31^ Layer 2 of Figure 1 depicts how researchers can examine how the lack of non-discrimination laws limit housing and employment options for transgender people, as was done in the Virginia Transgender Health Initiative Study.^55^ Additional questions include: How do cisgenderism, racism, and capitalism intersect to severely limit housing and education for transgender women of color? How do housing, education, and criminal**–**legal policies—at local, state, and national levels—interlock to cause racial inequities in incarceration, sex work or street economies, and in exchange of sex for food or housing? How do racialized gender norms influence the ways Black, Latina, and other young trans women of color are expected to look, act, and behave within society, thus creating an increased need for gender affirmation? Asking these types of questions will lead to specific interventions and intersectoral policies that could disrupt the structural production of transgender health inequities.

Naming intersecting power relations also prompts researchers to explain the meaning of their social variables, including race/ethnicity. Arayasirikul et al. situate the transphobic discrimination measures within a gender minority stress framework and they mention heteronormativity and heterosexism.^54^ However, they state an intention “to examine the effects of race on HIV risk factors” without explaining how they conceptualize “race”; apart from other explanations, this language of race effects is suggestive of biological determinism.^33,34^ Likewise, in their regression analysis of syndemics and social marginalization, Brennan et al. controlled for race/ethnicity, because race/ethnicity variables had “significant correlations with indicators of social marginalization.”^12^ IRTHJ emphasizes that readers of these articles recognize that race is a product of racialization processes^56^ and white supremacy. The IRTHJ framework also encourages researchers to theorize and diagram how race and racism interrelate causally with other study measures of social marginalization, instead of controlling for these social factors.

### Disrupt the status quo

All three papers focused on what the World Health Organization Commission on Social Determinants of Health (CSDH) has referred to as intermediary social determinants of health.^57^ A consequence of focusing at this level of causation is that the interventions proposed are likewise limited to action on intermediary determinants, which Solar and Irwin argue is insufficient: “Arguably the single most significant lesson of the CSDH conceptual framework is that interventions and policies to reduce health inequities must not limit themselves to intermediary determinants, but must include policies specifically crafted to tackle the social mechanisms that systematically produce an inequitable distribution of the determinants of health among population groups.”^58^

For example, Wilson et al. advocated for “[p]ublic health efforts that prioritize access to housing, education, and jobs” for transgender young women.^13^ IRTHJ encourages a focus, further upstream, on the specific policies and operational practices within and across institutional systems that result in the inequitable social patterning of housing, education, and jobs for transgender populations. Thus, the IRTHJ action of disrupting the status quo emphasizes action that eliminates transgender health inequities at the structural level. One example of a structural-level intervention is Hill et al., who explored employment among transgender women of color who had received no-cost legal name change support and found that those who had legally changed their name had greater odds of being employed.^59^ Supporting transgender people with accessible legal name changes can address the everyday marginalization that occurs within various institutional systems due to cisgenderism, thereby directly impacting social factors, such as housing and jobs that Wilson et al.^13^ identified were connected to HIV risk.

### Center embodied knowledge

Centering embodied knowledge asks researchers to use participatory methodologies that involve collaboration and power sharing with transgender community members throughout the research process. Unlike many observational studies, transgender research projects often include community members on advisory boards or in focus groups to guide study development. Wilson et al.^13^ used focus groups comprising transgender youth as part of their “formative assessment,” and Brennan et al.^12^ used a transgender advisory committee at one of their research sites to inform survey development, recruitment strategies, and study protocols. These practices allow for some inclusion of embodied knowledge in research design. However, IRTHJ calls for greater involvement of transgender community members, activists, and organizations—not only as advisors or key informants on study design, but also as full research collaborators with decision-making power over all aspects of the research from idea generation through dissemination. Local grassroots research organizations, such as Black Youth Project 100: Agenda to Build Black Futures provide examples of how to center marginalized voices and perspectives through action-oriented research that values embodied knowledge throughout the research process, including data analysis, dissemination, and action.^60^ Using models such as this, transgender health research can privilege the experiential knowledge of transgender people to promote strategic approaches for achieving transgender health justice.

## Conclusion

When researchers do not acknowledge the complex ways that power operates to create inequities in health outcomes, we are complicit in the making of structural injustice. Transgender health inequities research must instead move toward an agenda of health justice. IRTHJ is a novel, theory-driven conceptual framework to inform transgender health research that seeks to illuminate and disrupt structural causes of health inequities. Through application of IRTHJ actions, researchers can illuminate power hierarchies, bring an awareness of intersecting structural causes of health inequities to the research process and dissemination of findings, and work in solidarity with transgender populations to collectively seek to not only understand but also disrupt injustice and resulting inequities in health.

## Abbreviations Used

CRAI: condomless receptive anal intercourse
CSDH: Commission on Social Determinants of Health
IRTHJ: Intersectionality Research for Transgender Health Justice

## Notes

^‡^ We use the word transgender throughout this article as an umbrella term. We intend the term “transgender” to be inclusive of many diverse terminologies that people may use to describe themselves and the diversity of identities and experiences outside of the gender binary, including two-spirit, nonbinary, agender, and genderqueer people.
